# Core Microbiota and Metabolome of *Vitis vinifera* L. cv. Corvina Grapes and Musts

**DOI:** 10.3389/fmicb.2017.00457

**Published:** 2017-03-21

**Authors:** Irene Stefanini, Silvia Carlin, Noemi Tocci, Davide Albanese, Claudio Donati, Pietro Franceschi, Michele Paris, Alberto Zenato, Silvano Tempesta, Alberto Bronzato, Urska Vrhovsek, Fulvio Mattivi, Duccio Cavalieri

**Affiliations:** ^1^Computational Biology Department, Research and Innovation Centre, Edmund Mach FoundationSan Michele all'Adige, Italy; ^2^Food Quality and Nutrition Department, Research and Innovation Centre, Edmund Mach FoundationSan Michele all'Adige, Italy; ^3^Department of Agricultural, Food, Environmental and Animal Sciences, University of Udine, Via delle ScienzeUdine, Italy; ^4^Zenato Azienda Vitivinicola, Peschiera del GardaVerona, Italy; ^5^Center Agriculture Food Environment, University of TrentoSan Michele all'Adige, Italy

**Keywords:** *Vitis vinifera* L. cv. Corvina, volatile compounds, untargeted metabolomics, metataxonomics, fungal populations, grapes, musts

## Abstract

The composition and changes of the fungal population and of the metabolites present in grapes and in ferments of *Vitis vinifera* L. cv. Corvina, one of the major components of the Amarone musts, were dissected aiming at the identification of constant characteristics possibly influenced by the productive process. The fungal populations and metabolomic profiles were analyzed in three different vintages. 454-pyrosequencing on the ribosomal ITS1 region has been used to identify the fungal population present in Corvina grapes and fresh must. Samples were also subjected to metabolomics analysis measuring both free volatile compounds and glycosylated aroma precursors through an untargeted approach with comprehensive two-dimensional gas chromatography time-of-flight mass spectrometry. Albeit strongly dependent on the climate, both the mycobiota and metabolome of Corvina grapes and fresh musts show some characteristics recursive in different vintages. Such persistent characteristics are likely determined by the method adopted to produce Amarone or other dry wines made from partially dried grapes. In particular, the harsh conditions imposed by the prolonged withering appear to contribute to the shaping of the fungal populations. The fungal genera and metabolites present in different vintages in *V. vinifera* L. cv. Corvina grapes and fresh musts represent core components of the peculiar technique of production of Amarone. Their identification allows the in-depth understanding and improved control of the process of production of this economically and culturally relevant wine.

## Introduction

Amarone is a dry wine produced exclusively in the Italian region of Valpolicella (Verona) by the combination of *Vitis vinifera* L. cv. Corvina and *V. vinifera* L. cv. Rondinella withered red grapes (45–95% Corvina, 5–30% Rondinella, Paronetto and Dellaglio, 2011). Since 2010 Amarone is a DOCG (Denominazione di Origine Controllata e Garantita, “Controlled and Guaranteed Denomination of Origin”) wine, a category reserved for the highest quality wines from Italy, thus its production is subjected to a strict regulation (reviewed by Paronetto and Dellaglio, 2011). Corvina grapes, composing the main part of the Amarone must, have been shown to hold a fundamental role in conferring the organoleptic characteristics to the wine (Di Carli et al., 2011; Fedrizzi et al., 2011; Toffali et al., 2011). As an additional step characterizing the Amarone production, grapes are subjected to a long withering period. Grapes harvested at ripening are stored in well-aired warehouses until they lose up to 40% of their initial mass (Williams et al., 1989). During this process, which can last up to 2–3 months due to the relatively low environmental temperatures of the late autumn/early winter, grapes are dried and increase their sugar content up to about 30% (Consonni et al., 2011). Several metabolites relevant for the aromatic bouquet of wine have been shown to evolve in this phase of the process (Consonni et al., 2011; Fedrizzi et al., 2011; Paronetto and Dellaglio, 2011). The withering process is also known to favor the growth of fungi of the genus *Botrytis*, the “noble rot” whose relevance in determining the microbial and chemical characteristics of withered wines has been suggested (Fedrizzi et al., 2011; Consonni et al., 2011; Bokulich et al., 2012). In a recent study, Salvetti and co-workers have shown that the microbial populations present after the grape withering is widely defined by drying parameters such as temperature, relative humidity, and ventilation (Salvetti et al., 2016). Nevertheless, other factors have been shown to severely affect the microbial populations present on grape skins and thus in the early phases of must fermentation. Among these, the environmental changes associated to the vintage, climate, geography, and cultivar (Bokulich et al., 2014). Aiming at the disclosure of the influence of the productive process on the final product regardless to other environmental parameters, we studied the changes in microbial populations and in the metabolome of grapes of Corvina during and after the withering period. As a case-study, we analyzed grapes and musts sampled from the warehouse of Cantina Zenato, located in Valpolicella, the Italian viticultural zone where the grape varieties used for Amarone production (Corvina and Rondinella) are typically produced. To assess whether the influence of the productive process was constant across different years, we compared the fungal populations and metabolome among vintages characterized by both standard (the 2013 and 2015 vintages) and extreme climates (the 2014 vintage, characterized by abnormal, and abundant rainfalls). As a consequence of these peculiar atmospheric conditions, the wine-makers adopted extraordinary approaches in the vineyard (with repeated treatments with antifungals), in deciding the day of grape harvesting (which has to be done in absence of precipitations) and in the following steps of the wine-making process. Considering this, the inclusion in our study of this vintage, unfortunate by the wine-maker economic viewpoint, gave us the opportunity to observe the natural behavior of fungal communities in an extremely wet year, representing an interesting “case study.”

## Materials and methods

### Sampling

Samples were collected from the warehouse of Cantina Zenato, located in Valpolicella, the Italian viticultural zone located in the province of Verona (Italy), where red wine is typically made from three grape varieties: Corvina (*V. vinifera* L. cv. Corvina), Rondinella, and Molinara. Grapes and musts were sampled during three vintages (2013–2015). The time of harvesting was decided by the wine-makers according to the evaluated grade of grape maturation. After the harvesting in the vineyard “Costalunga” (also located in the Valpolicella region, Sant'Ambrogio, Verona, Italy), grapes were subjected to withering in a dedicated warehouse located a few kilometers far from the vineyards (<5 Km). The warehouse was equipped with automatic systems able to control and modify the internal temperature and humidity. The duration of grape withering was defined according to the regional rules for Amarone production (reviewed in Paronetto and Dellaglio, 2011), which defines the time in which the grapes for Amarone vinification can be mashed. In light of this, the withering period varied in the 3 years of study (71 days in 2013, 107 days in 2014, 77 days in 2015). Grapes were then mashed in the warehouse used for the withering and fermentations were carried out in stainless steel tanks. Grapes were collected at two time-points during the withering period. The first grape sampling (T0) occurred a few hours after the harvesting, once the grapes were transferred from the vineyard to the warehouse. The second sampling occurred after 7–8 weeks since the start of the withering (58th, 49th, and 63rd day after harvesting in the 2013, 2014, and 2015 vintages, respectively). For each time-point of grapes sampling, six biological replicates were collected in the 2013 vintage and eight in the 2014 vintage (a bunch for each replicate). Grapes were mashed at the end of the withering period, namely at the 71st, 107th, and 77th day after harvesting in the 2013, 2014, and 2015 vintages, respectively (Table 1). Musts samples were collected as soon as the grapes were mashed. To ensure sampling representativity musts were mixed with sterile tools before sampling. In addition, for each sampling equal amounts were sampled from the bottom, from the middle and from the top of the tank, then mixed again and analyzed as an unique sample. Two must samples were collected in 2013 and 2015 vintages, eight in 2014. Samples were collected in sterile tubes then stored at −80°C until DNA extraction. A summary of samples details is shown in Table 1.

**Table 1:** **Samples metadata**.

**Sample_name**	**Year**	**Sample matrix**	**Sampling date (dd/mm/yy)**	**Time from harvesting (days)**	Submitted data ID^*^	
					**Meta Taxonomics**	**Metabolomics**
**2013 VINTAGE. HARVESTING DATE: 25/09/13, BEGIN OF FERMENTATION: 05/12/13**						
X2B	2013	Grape	25/09/13	0	ERS1554447	NA
X3B	2013	Grape	25/09/13	0	ERS1554446	NA
X4B	2013	Grape	25/09/13	0	ERS1554448	NA
X5B	2013	Grape	25/09/13	0	ERS1554449	NA
Y1B	2013	Grape	25/09/13	0	ERS1554450	NA
Y5B	2013	Grape	25/09/13	0	ERS1554451	NA
X1	2013	Grape	23/11/13	58	ERS1303807	NA
X2	2013	Grape	23/11/13	58	ERS1303808	NA
X6	2013	Grape	23/11/13	58	ERS1303809	NA
Y1	2013	Grape	23/11/13	58	ERS1303810	NA
Y2	2013	Grape	23/11/13	58	ERS1303811	NA
Y6	2013	Grape	23/11/13	58	ERS1303812	NA
5F6	2013	must	05/12/13	71	ERS1303814	NA
5F8	2013	must	05/12/13	71	ERS1303813	NA
**2014 VINTAGE. HARVESTING DATE: 23/09/14, BEGIN OF FERMENTATION: 08/01/15**						
C14Fr1G1A	2014	Grape	23/09/14	0	ERS1554456	NA
C14Fr1G2A	2014	Grape	23/09/14	0	ERS1554457	NA
C14Fr1G3A	2014	Grape	23/09/14	0	ERS1554458	NA
C14Fr1G4A	2014	Grape	23/09/14	0	ERS1554459	NA
C14Fr1C1A	2014	Grape	23/09/14	0	ERS1554452	NA
C14Fr1C2M	2014	Grape	23/09/14	0	ERS1554453	NA
C14Fr1C3M	2014	Grape	23/09/14	0	ERS1554454	NA
C14Fr1C5M	2014	Grape	23/09/14	0	ERS1554455	NA
C14Fr2G1A	2014	Grape	11/11/14	49	ERS1303818	MTBLS392- C14Fr2G1A
C14Fr2G2A	2014	Grape	11/11/14	49	ERS1303819	MTBLS392-C14Fr2G2A
C14Fr2G3A	2014	Grape	11/11/14	49	ERS1303820	MTBLS392-C14Fr2G3A
C14Fr2G4A	2014	Grape	11/11/14	49	ERS1303822	MTBLS392-C14Fr2G4A
C14Fr2C1A	2014	Grape	11/11/14	49	ERS1303815	MTBLS392-C14Fr2C1A
C14Fr2C2A	2014	Grape	11/11/14	49	ERS1303816	MTBLS392-C141Fr2C2A
C14Fr2C3A	2014	Grape	11/11/14	49	ERS1303817	MTBLS392-C14Fr2C3A
C14Fr2C5A	2014	Grape	11/11/14	49	ERS1303821	MTBLS392-C14Fr2C5A
CP1_T0	2014	Must	08/01/15	107	ERS1303826	MTBLS392-CP1_T0
CP2_T0	2014	Must	08/01/15	107	ERS1303827	MTBLS392-CP2_T0
CP3_T0	2014	Must	08/01/15	107	ERS1303828	MTBLS392-CP3_T0
CM1_T0	2014	Must	08/01/15	107	ERS1303823	MTBLS392-CM1_T0
CM2_T0	2014	Must	08/01/15	107	ERS1303824	MTBLS392-CM2_T0
CM3_T0	2014	Must	08/01/15	107	ERS1303825	MTBLS392-CM3_T0
LP1_T0	2014	Must	08/01/15	107	ERS1303832	MTBLS392-LP1_T0
LP2_T0	2014	Must	08/01/15	107	ERS1303833	MTBLS392-LP2_T0
LP3_T0	2014	Must	08/01/15	107	ERS1303834	MTBLS392-LP3_T0
LM1_T0	2014	Must	08/01/15	107	ERS1303829	MTBLS392-LM1_T0
LM2_T0	2014	Must	08/01/15	107	ERS1303830	MTBLS392-LM2_T0
LM3_T0	2014	Must	08/01/15	107	ERS1303831	MTBLS392-LM3_T0
**2015 VINTAGE. HARVESTING DATE: 10/09/15, BEGIN OF FERMENTATION: 12/11/15**						
216A	2015	Must	12/11/15	63	NA	MTBLS392-216A
216B	2015	Must	26/11/15	77	NA	MTBLS392-216B
224A	2015	Must	12/11/15	63	NA	MTBLS392-224A
224B	2015	Must	26/11/15	77	NA	MTBLS392-224B

* *Meta-taxonomics data were submitted to the European Nucleotide Archive with project accession number PRJEB15229, metabolomics data were submitted to the MetaboLights database with accession number MTBLS392. NA = the sample was not analyzed with the corresponding technique*.

### DNA extraction

Aiming at the quantification of the total amounts of microbial populations, microbial DNA was extracted from both grape and must samples collected in all the studied vintages. DNAs extracted from the 2013 and 2014 vintages samples were also used for meta-taxonomic analyses. Extraction of DNA was carried out from 4 grapes or 2 ml thawed must. While grapes were directly subjected to microbial DNA extraction, must samples were subjected to a prior treatment to remove substances (i.e., polyphenols) which could interfere with the DNA extraction. Musts were centrifuged 30 min at 14,000 g and at 4°C, and the pellet was dissolved in 2 ml TE buffer. Must was centrifuged again for 15 min at 14,000 g at 4°C, and the pellet was dissolved in 300 μl TE buffer. Extraction of DNA was then carried out with the FastDNA Spin Kit for Soil (MP biomedicals) following the manufacturer's instructions.

### Quantification of total fungi and bacteria

To monitor the absolute amounts of microbes present in the samples, we carried out quantitative Real Time PCR (qRT-PCR) as previously described (Stefanini et al., 2016). Total bacterial and fungal DNAs extracted from all the matrices collected in all the studied vintages were quantified by using universal primers specific for either the V1–V3 region of 16S rRNA gene for bacteria (Baker et al., 2003), or the ITS1 region for fungi (Findley et al., 2013). Real-time PCR was performed with a LightCycler(R) 480 (Roche) using optical grade 96-well plates. The PCR reaction was performed in a total volume of 12.5 μl using the KAPA SYBR(R) Fast qPCR Kit (KAPABiosystems) as previously described (Stefanini et al., 2016). Wilcoxon-Mann-Whitney test was carried out to compare the total amount of either fungi or bacteria in different groups of samples.

### Meta-taxonomic 454-pyrosequencing and data analysis

Meta-taxonomics analysis was carried out on samples collected in the 2013 and 2014 vintages. Library preparation, sequencing, and initial raw sequences processing were carried out by the Sequencing Platform at Fondazione E. Mach. Fungal genera identification was carried out by mean of 454-pyrosequencing of the ITS1 region. To avoid biases in abundance detection due to preferential amplifications of sequences showing different lengths (ITS1-5.8S-ITS2), we sequenced the ITS1 region which is not subjected to the wide length polymorphism affecting the entire ITS region (Op De Beeck et al., 2014). For each sample, fungal ITS1 rDNA region was amplified using a specific fusion primer set coupled with forward primer 18SF (5′-GTAAAAGTCGTAACAAGGTTTC-3′) and reverse primer 5.8S1R (5′-GTTCAAAGAYTCGATGATTCAC-3′; Findley et al., 2013) containing adaptors, key sequence and barcode (Multiple IDentifier) sequences as described by the 454 Sequencing System Guidelines for Amplicon Experimental Design (Roche, Switzerland). The forward primer sequences were made up of: the “LIB-L” primer A sequence specific for “Lib-L” chemistry and “One-Way Reads” sequencing methods (Roche, Branford, CT), the key sequence TCAG, the bar code MID (Multiple IDentifier) sequence specific for any sample and the forward primer sequence. The reverse primer contained the “Lib-L” primer B sequence, the key sequence TCAG and the reverse primer sequence. For each sample, a PCR mix of 25 μl was prepared containing 1X PCR buffer, 1.25 U of FastStart High Fidelity polymerase blend (Roche) and dNTPs from the FastStart High Fidelity PCR system (Roche), 0.4 μM of each primer (PRIMM, Milano) and 10 ng of gDNA. Thermal cycling consisted of initial denaturation at 94°C for 3 min followed by 25 cycles (35 for fungal ITS) of denaturation at 94°C for 15 s, annealing at 60°C (58°C for fungal ITS) for 45 s, and extension at 72°C for 1 min, with a final extension of 8 min at 72°C. Products of PCR were analyzed through gel electrophoresis and cleaned using the AMPure XP beads kit (Beckman Coulter, Brea, CA, USA) following the manufacturer's instructions. Products of the different samples were quantified via quantitative PCR using the Library quantification kit—Roche 454 titanium (KAPA Biosystems, Boston, MA) and pooled in an equimolar way in a final amplicon library. 454-pyrosequencing was carried out on the GS FLX + system using XL + chemistry, following the manufacturer's recommendations. Pyrosequencing produced a total of 31,4559 reads of ITS1 region (for 28 sequenced samples). The sequences were assigned to samples according to sample-specific barcodes. This allowed us to collect FASTA formatted files containing an average of 11234.25 ± 3132.04 (*SD*) sequences per sample. Sequences were then checked for the following criteria: (i) no more than one mismatch/deletion/insertion both in the bar code and in the primer, (ii) length of at least 150 nucleotides (barcodes and primers excluded) and (iii) no more than two undetermined bases (denoted by N). Data were submitted to the European Nucleotide Archive with accession number PRJEB15229 (http://www.ebi.ac.uk/ena/data/view/PRJEB15229). The correspondence between submitted data IDs and samples is shown in Table 1.

Raw data files generated by the Roche 454 sequencer were de-multiplexed using Roche's sfffile software. Reads were pre-processed using the micca pipeline v0.1 (Albanese et al., 2015). Forward and reverse primers trimming and quality filtering were performed using micca-preproc (parameters -f GTTTCCGTAGGTGAACCTGC -r TCCTCCGCTTATTGATATGC -O 16 -l 150 -q 18), truncating reads shorter than 150 bp. *De novo* sequence clustering, chimera filtering and taxonomy assignment were performed using micca-otu-denovo (parameters –s 0.97 –c): OTUs were assigned by clustering the sequences with a threshold of 97% pairwise identity, and their representative sequences were classified using blast against the “unite” database (Kõljalg et al., 2013) (release 09/02/2014). For ITS data, multiple sequence alignment (MSA) and phylogenetic tree inference were performed using the online version of T-Coffe (Notredame et al., 2000). The taxonomies of all the representative sequences identified in the samples were further checked by manually blasting each sequence through the National Center for Biotechnology Information nucleotide collection database (http://blast.ncbi.nlm.nih.gov/Blast.cgi?PROGRAM=blastn&PAGE_TYPE=BlastSearch&LINK_LOC=blasthome). Blasting results are shown in Supplementary Table 1. Sampling heterogeneity was reduced by rarefaction (2,500 sequences per sample). Alpha (within-sample richness) and beta-diversity (between-sample dissimilarity) estimates were computed using the phyloseq r package (McMurdie and Holmes, 2013). Two-sided, unpaired Welch *t*-statistics were computed using the function mt() in the phyloseq library (McMurdie and Holmes, 2013), and the *p*-values were adjusted for multiple comparison controlling the familywise Type I error rate (minP procedure) (Westfall and Young, 1993). PERMANOVA (Permutational multivariate analysis of variance) was performed using the adonis() function of the *vegan* R package with 999 permutations. Correlations among fungal species and chemicals were evaluated with the psych r package (Revelle, 2015). False discovery rate (FDR)-adjusted *P*-values were computed using the Benjamini–Hochberg procedure (Benjamini and Hochberg, 1995).

### Metabolomics measurement and analysis

Metabolomics analysis was carried out for all the samples collected in the 2014 and 2015 vintages. Free and bound (glycosylated aroma precursors) volatile organic compounds (VOCs) were extracted following the SPE method reported in Vrhovsek et al. (2014). Extracts were injected using a Gerstel MultiPurpose Sampler autosampler (Gerstel GmbH & Co. KG Mülheim an der Ruhr Germany) into a comprehensive two-dimensional gas chromatography time-of-flight mass spectrometry (GC × GC-MS) system consisting of an Agilent 7890 A (Agilent Technologies, Santa Clara, CA) equipped with a Pegasus IV time-of-flight mass spectrometer (Leco Corporation, St. Joseph, MI). A VF-Wax column (100% polyethylene glycol; 30 m × 0.25 mm × 0.25 μm, Agilent J&W Scientific Inc., Folsom, CA) was used as first-dimension (1D) column, and a Rxi-17Sil MS-column (Restek Bellefonte, USA) (mid polarity phase) 1.50 m × 0.15 mm × 0.15 μm, (Restek Bellefonte, USA) was used as a second-dimension (2D) column. The GC system was equipped with a secondary column oven and non-moving quadjet dual-stage thermal modulator. The injector/transfer line was maintained at 250°C. Oven temperature programme conditions were as follows: initial temperature of 40°C for 4 min, programmed at 6°Cmin^−1^ at 250°C, hold for 5 min. The secondary oven was kept 5°C above the primary oven throughout the chromatographic run. The modulator was offset by +15°C in relation to the secondary oven; the modulation time was 7 and 1.4 s of hot pulse duration. Helium (99.9995% purity) was used as carrier gas at a constant flow of 1.2 mL min-1 The MS parameters included electron ionization at 70 eV with ion source temperature at 230°C, detector voltage of 1317 V, mass range of m/z 35–450 and acquisition rate of 200 spectra s^−1^. For GC × GC-MS data a LECO ChromaTOF (Version 4.22) software was used for acquisition control, and data processing. The identification of volatile compounds was done using NIST 2.0, Wiley 8, and the FFNSC 2 mass spectral library (Chromaleont, Messina, Italy), with a library similarity match factor of 750. Raw metabolomic data were submitted to the MetaboLights database with accession number MTBLS392. Free and glycosylated aroma precursors volatile organic compounds were annotated by comparing their mass spectra to NIST 2.0, Wiley 8, and FFNSC 2 libraries, with a similarity match factor of 750. Compounds with a similarity match factor lower than 750 were classified as “unknown”. In the 2014 vintage samples, 93 glycosylated aroma precursors (89 tentatively identified + 4 unknown) and 728 free compounds (664 tentatively identified + 64 unknown) were measured. In the must samples collected in the 2015 vintage 947 free volatile compounds were measured. The response of internal standard 1-heptanol was used for normalization and to make a relative estimation of the identified compounds as commonly accepted in the analysis of aroma compounds. Wilcoxon-Mann-Withney test was carried out to evaluate the significance in the differences abundance of each chemical among either sample types (grapes or musts) or vintages. Pearson coefficients were calculated between chemical compound content and fungal genera relative abundances. All the calculated *p*-values were adjusted for multiple comparison (FDR) (Westfall and Young, 1993).

## Results

### Climate and withering effects on the mycobiota diversity

**Figure 1:**
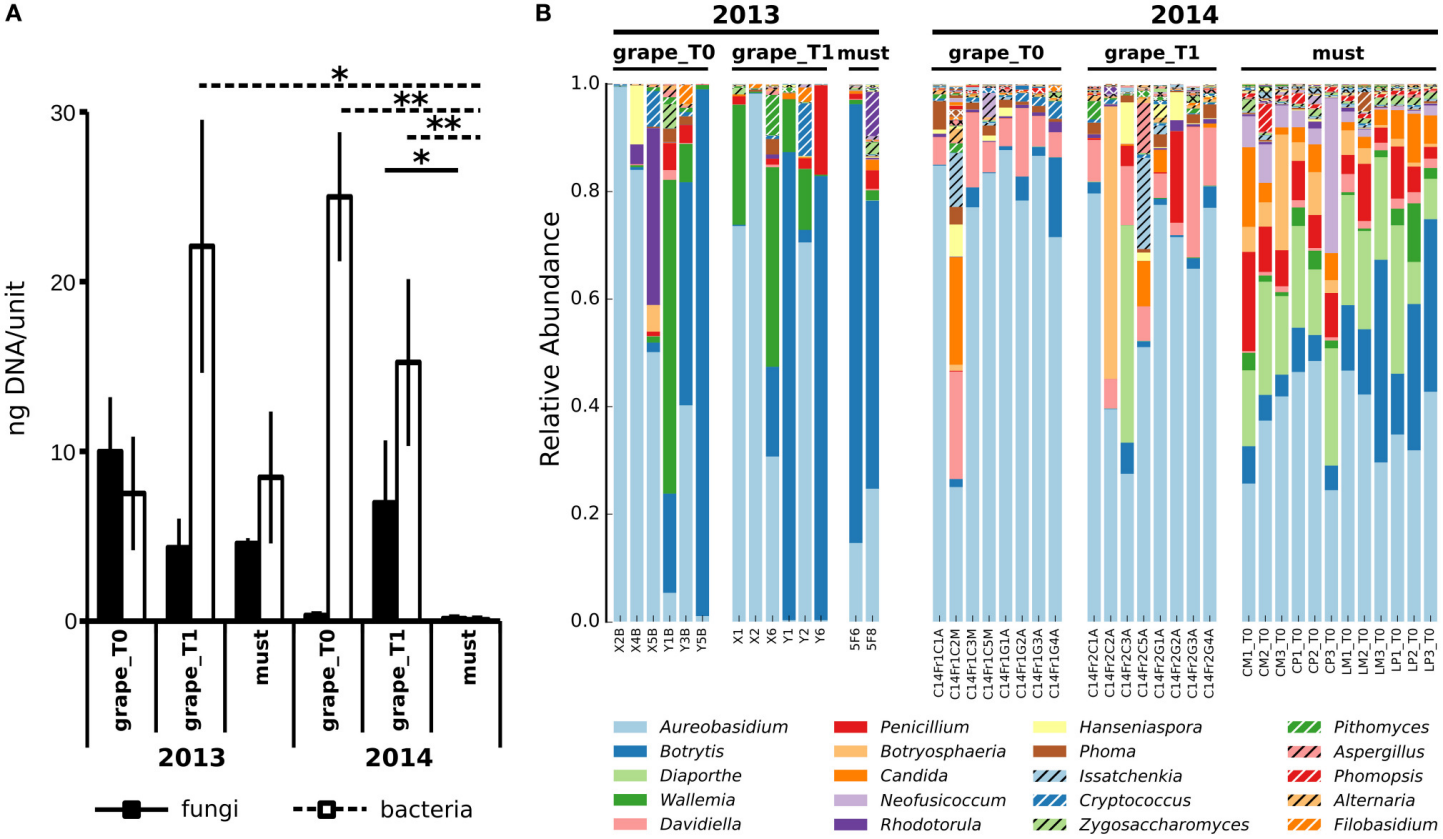
**Microbial populations in Corvina grapes and fresh musts. (A)** Total amount of fungal and bacterial DNAs, estimated by mean of qRT-PCR based on the ITS1 and 16S ribosomal sequences for fungi and bacteria, respectively. Values indicate the amount of fungal or bacterial DNA quantified in 4 grapes or 2 ml of musts per sample. ^*^Wilcoxon-Mann-Whitney FDR < 0.05, ^**^Wilcoxon-Mann-Whitney FDR < 0.01. **(B)** Composition profiles of Vitis vinifera L. cv. Corvina grapes and musts mycobiota. Relative abundance of operational taxonomic units (OTUs) from fungal species in Vitis vinifera L. cv. Corvina grape and must samples. Colors correspond to genera, the 20 most abundant genera are listed in the figure legend.

**Figure 2:**
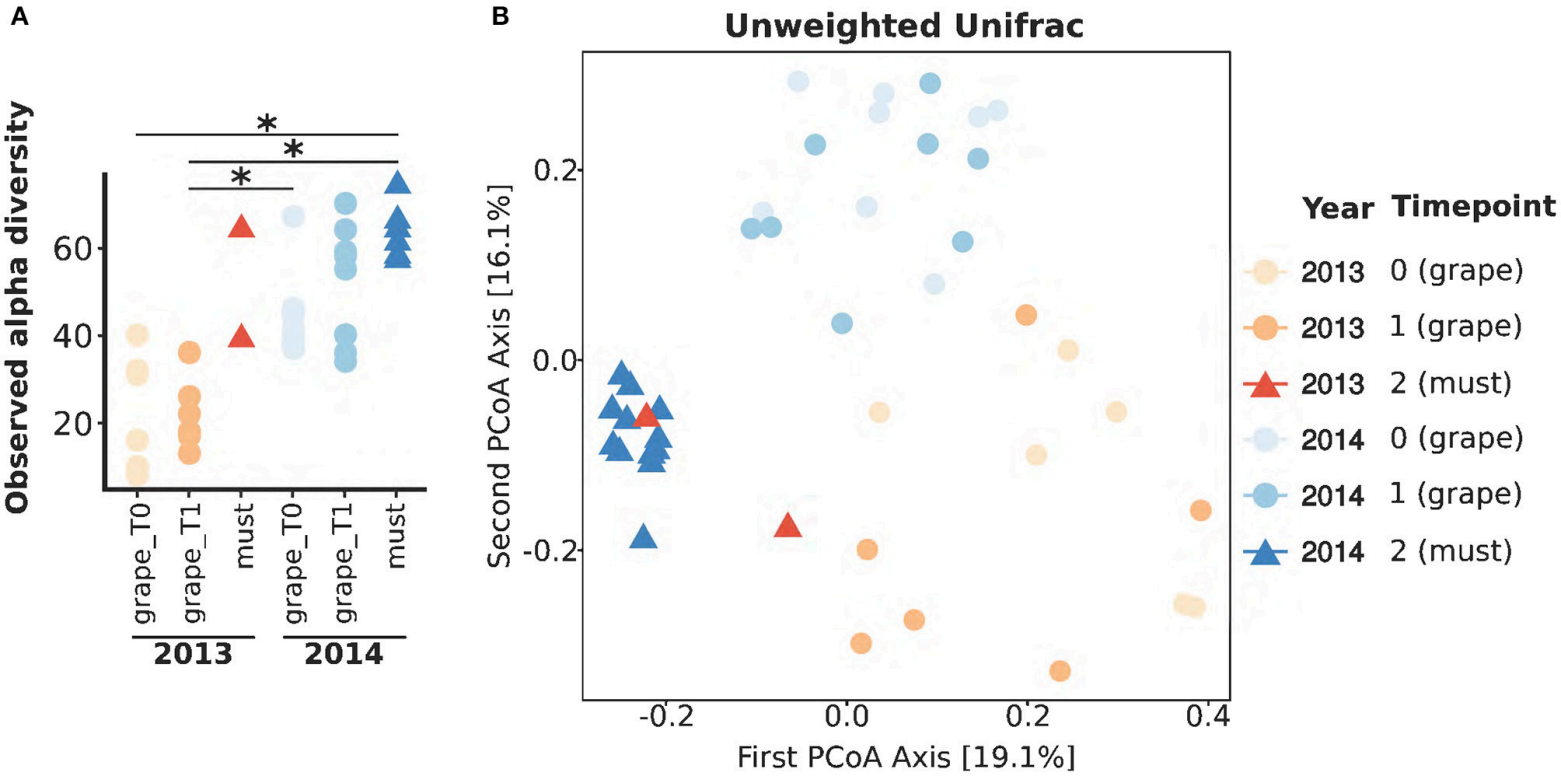
**Fungal population diversities in Corvina grapes and fresh musts. (A)** Observed OTUs of fungal populations present in grape and must samples. ^*^Wilcoxon-Mann-Whitney FDR < 0.05. **(B)** First two coordinates if the Principal Coordinates Analysis (PCoA) carried out on samples distances calculated with the unweighted UniFrac metric.

To compare the populations of grapes and musts sampled in different vintages, we first sought to compare their general characteristics. Aiming at this, the total amounts of fungi and bacteria were quantified in *Vitis vinifera* L. cv Corvina grapes and must samples collected in two vintages, 2013 and 2014, by mean of quantitative Real Time PCR (qRT-PCR) (Figure 1A). Grapes were sampled at two timepoints: T0, when grapes were transferred in the warehouse, and T1, after 7–8 weeks of withering (see Table 1 for details). Must samples were collected right after grape mashing at the end of the withering period, whose length varied in different vintages (Table 1). The slow withering process imposed environmental stresses to the grapes microbiota, potentially selecting the tolerant bacteria, and fungi, we thus expected to find significant variations in the total amounts of bacteria and fungi. Noteworthy, despite widely changing during the process, neither the total amount of fungi nor the total amount of bacteria significantly changed during the initial withering (grape_T0 versus grape_T1) in any of the studied vintages (Figure 1A). In addition, in the first sampled vintage (2013), the amounts of total bacteria and fungi did not differ between grape and fresh must samples. Contrarily, the total amount of bacteria decreased from the beginning to the end of the 2014 withering, being it significantly lower at each sampled time-point (Wilcoxon-Mann-Whitney FDR < 0.05, Figure 1A and Supplementary Table 2). To note, the fungal total amount significantly changed only between grapes sampled at the second withering time-point and fresh musts in 2014 vintage (Wilcoxon-Mann-Whitney FDR < 0.05, Figure 1A). To further explore the differences in microbial populations present in Corvina grapes and musts in the two vintages, we carried out 454-pyrosequencing meta-taxonomics analysis based on fungal ITS1 region sequencing aiming at the identification of the fungal genera present in the samples. We were able to describe at the genus level the fungal populations present in the samples (OTUs relative abundances are shown in Figure 1B). Differences between fungal populations were observable by comparing the genera relative abundances (Figure 1B), highlighting wide differences between samples of the different vintages and similarities between grape time-points of the same vintage, while slight differences were observable between grapes and fresh musts in both vintages. We went further and quantified the changes by comparing alpha (within sample) and beta (between samples) diversities. As observed when comparing the total amounts of fungi and bacteria, the observed number of OTUs did not differ between grapes sampled at the two time-points in any of the studied vintages (Figure 2A). In addition, both time-points of grapes sampled in 2013 bore a lower number of fungal genera than the 2014 must samples (Wilcoxon-Mann-Whitney FDR = 0.028 for both comparisons, Supplementary Table 2), but no significant differences were found between different sample types (grapes and musts) of the same vintage (Figure 2A). The same situation was observable by comparing Chao1 and Shannon indexes of alpha diversity (Supplementary Figure 1). On the other hand, the beta diversity analysis (in terms of unweighted UniFrac distance) showed that the samples were significantly grouped according to the vintage (PERMANOVA corrected for nested variables, FDR = 0.001) but also to the sample type (i.e., grapes vs. musts, PERMANOVA corrected for nested variables, FDR = 0.001) (Figure 2B). No significant differences were found between grapes at the beginning of the withering period (T0) and T1 in the same vintage (Supplementary Table 2). In addition, while neither grapes collected at T0 nor grapes collected at T1 in 2013 showed significant differences compared to the must of the same year, both grapes timepoints significantly differed from musts in 2014 (PERMANOVA FDR < 0.05, Supplementary Table 2). The same results were observed by using two other widely used beta diversity measures, namely the Bray-Curtis and weighted UniFrac beta dissimilarities (Supplementary Table 2 and Supplementary Figure 2).

### The corvina mycobiota: effects of the climate and constant genera

**Figure 3:**
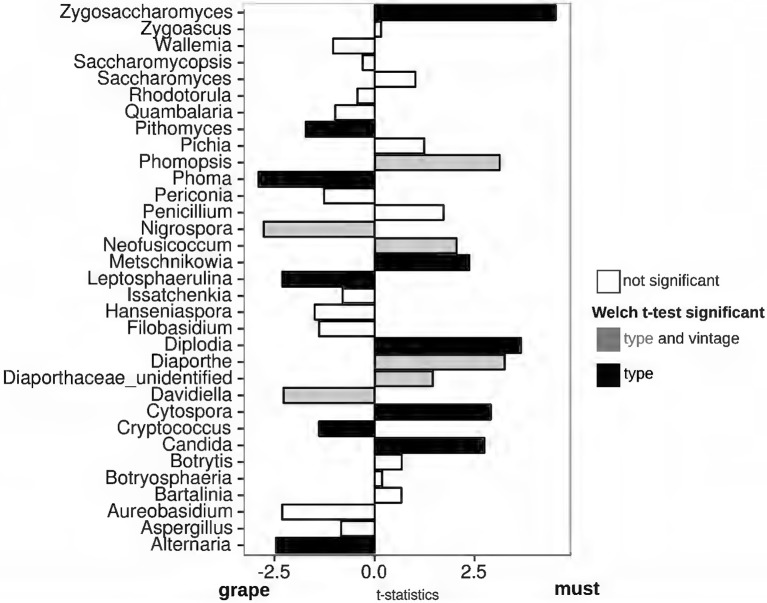
**Welch's t-statistics comparing the relative abundances of fungal genera present in grapes and must samples**. Welch's *t*-test was carried out to compare the relative abundances of fungal genera in grape and must samples. Black = FDR < 0.05 among sample matrices and FDR > 0.05 among vintages, Gray = FDR < 0.05 among matrices and FDR < 0.05 among vintages, white = FDR > 0.05.

By comparing fungal populations of grape and must samples during different vintages, we could identify the fungal genera whose presence was modified by the withering process, irrespective of the seasonal changes. As observed with other parameters (see previous section), the grape samples collected at T0 and at T1 were substantially similar even when comparing the fungal genera relative abundances (Welch *t*-test FDR > 0.05, Supplementary Figure 3). To note, despite the wide similarity among grapes at T0 and T1, the effect of the vintage on fungal genera relative abundances was not identical (Supplementary Figure 4). Indeed, the relative abundances of Sporobolomyces, Periconia, Peniophora, Leptosphaerulina, Issatchenkia, Bathalimia, Diaporthe, and Botrytis differed in T0 grapes harvested in 2014 and in 2013 (Welch *t*-test FDR < 0.05, Supplementary Figure 4) and did not differ in abundances in T1 samples of the two vintages. Grapes sampled at T1 were shown by several parameters to be indistinguishable from samples collected at T0 in the same vintage, suggesting that the withering affects fungal populations after prolonged periods. To identify the changes triggered by the withering on genera fungal abundances, we thus compared grapes sampled at T1 and fresh musts. Several genera were identified to be more abundant in grape than in must samples, without abundance differences between the vintages (Figure 3). The fungal genera *Pithomyces, Phoma, Leptospherulina, Cryptococcus*, and *Alternaria* were significantly more abundant in grape than in must samples (Welch *t*-test FDR < 0.05, Figure 3). *Vice*-*versa*, must samples showed a higher amount of the genera *Zygosaccharomyces, Metschnikowia, Diplodia, Cytospora*, and *Candida*, compared to grape samples (FDR < 0.05, Figure 3). In particular, *Diplodia* was found exclusively in must samples (Supplementary Figure 5). Considering the known problems in taxonomic assignments of species belonging to the Candida and Metschnikowia genera, we further evaluated our classification by clustering the representative sequences found in our samples with the Candida spp. and Metschnikowia spp. sequences deposited in the UNITE database (Supplementary Figure 6). Also other fungal genera were differentially represented in the two matrices, but the differences were strongly influenced by seasonal changes (Supplementary Figure 4). As an example, the *Davidiella* (*Cladosporium*, Bensch et al., 2012) and *Nigrospora* genera were significantly more abundant in all the samples collected in the 2014 vintage (Supplementary Figure 4), and were estimated to be more abundant in grape samples compared to must (Figure 3). Other examples of this situation are shown by the fungal genera *Phomopsis, Neofusicoccum, Diaporthe*, and *Diaporthe_unidentified*, which were significantly more abundant in must samples, but whose presence was significantly higher in the 2014 vintage. To assess whether the genera enriched in grapes and musts were mutually exclusive (the presence of one corresponds to the absence of another one or vice-versa), we calculated the Pearson coefficient between every possible couple of genera enriched in grapes and must samples. As expected, strong positive correlations were found among fungal genera that were enriched in the same matrix (grape or must) (Supplementary Figure 7). When observing the correlations between genera more abundant in musts and those more abundant in grapes, we found negative correlations among: (i) *Zygosaccharomyces* and *Metschnikowia* with genera more abundant in grapes, (ii) *Candida* (must) and *Leptosphaerulina* (grape) and (iii) *Cytospora* (must) and *Alternaria* and *Pithomyces* (grape) (Supplementary Figure 7).

### The corvina metabolome: relations with the climate

To further describe the process of Amarone production from the microbial viewpoint, we explored the metabolomic profiles of *V. vinifera* L. cv. Corvina grapes and musts in different vintages by mean of untargeted analysis (Versini et al., 2008). We assessed the contribution of grape withering in modifying the wine metabolome by comparing free and glycosylated precursor compounds in grapes and musts (before and after the withering) collected in the same vintage (2014). In addition, we evaluated which part of the chemical profiles were conserved in different vintages by comparing the free compounds (the most relevant for the wine flavor) in musts collected in two different vintages (2014 and 2015) (Michlmayr et al., 2012). Because grapes sampled at T1 were shown to be indistinguishable from samples collected at T0 in the same vintage, and aiming at the minimization of the matrix effect on metabolite measurement (grapes at drier at T1 than at T0, then more similar to musts) we carried out the following analyses by using the latter only and comparing them to must samples. To evaluate the effect of the withering process, we compared the relative amounts of free and glycosylated precursor compounds in grapes (before withering) and fresh musts (after withering) sampled in the 2014 vintage. Among the free compounds, 11 were significantly more abundant in must than in grape samples and 170 were showing an opposite trend (Supplementary Table 3). In terms of glycosylated aroma precursors, none was more abundant in grape than in must samples, while, vice-versa, 67 of them were significantly more abundant in musts (Supplementary Table 4). A similar increase in glycosylated precursor compounds was previously observed to occur during grape maturation (Ryona and Sacks, 2013), thus suggesting that the processes begun in grape ripening proceed also during withering as previously proposed (Di Carli et al., 2011). Beyond that, the relative amount profiles suggest a very effective extraction of the highly polar and water-soluble bound precursors from the skins into the juices.

**Figure 4:**
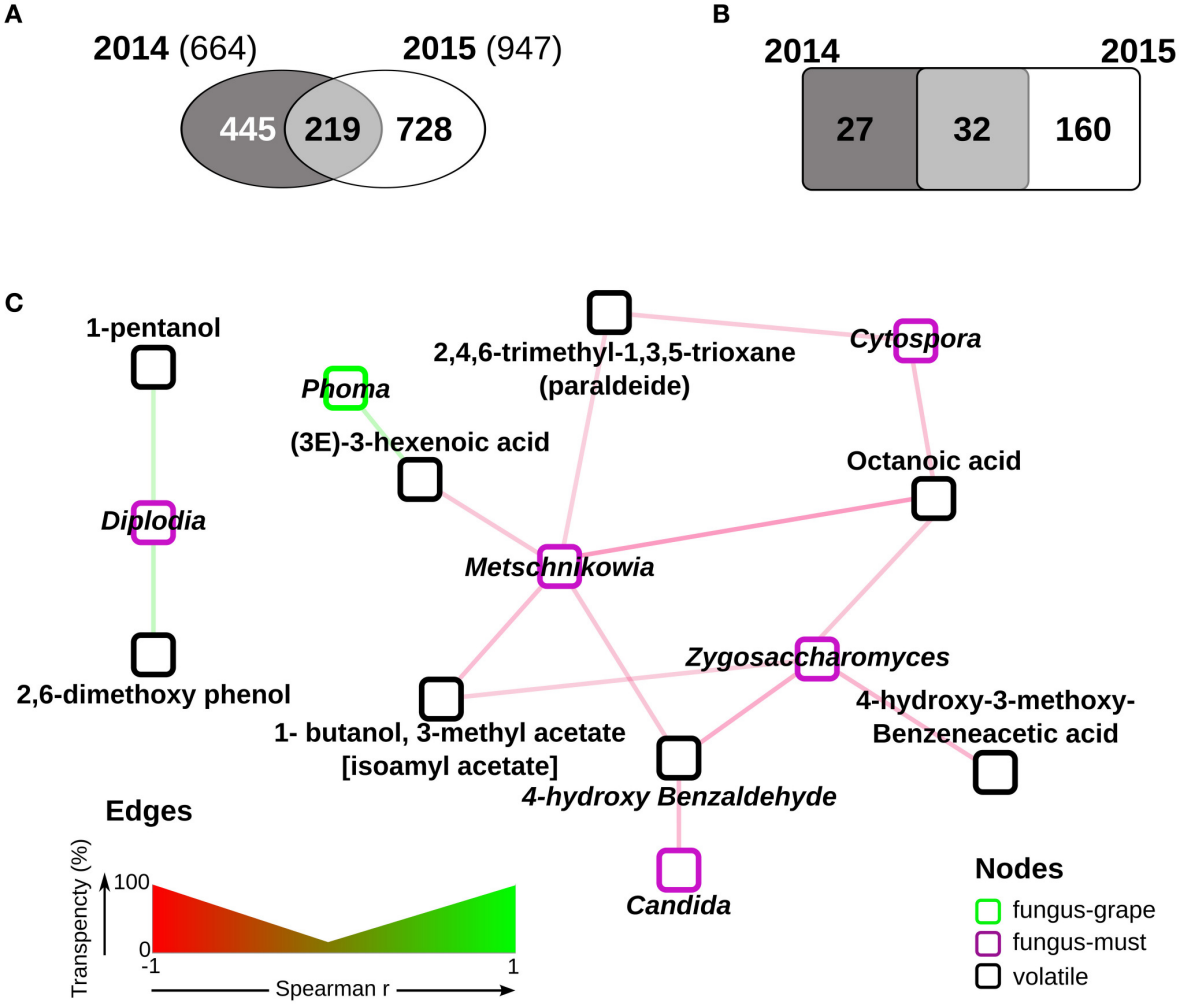
**The Corvina volatolome. (A)** comparison of free compounds present in must samples of the two vintages. 219 compounds were found in both the 2014 and 2015 samples; **(B)** comparison of the relative amounts of the 219 free compounds measured in both vintages. Free compounds were considered significantly enriched in either the 2014 or the 2015 samples when Wilcoxon-Mann-Whitney FDR < 0.05; **(C)** correlations among free compounds and fungal genera found in Corvina samples. Significant Pearson correlations (FDR < 0.05) were calculated among the free compounds and the fungal genera measured in must samples at similar relative amounts in the two vintages. The network was drawn with Cytoscape 3.4 (Shannon et al., 2013) with the significant correlations only.

We then focused on the free compounds consistently present in 2014 and 2015, since they directly influence the aroma characteristics. It is worth mentioning that even if the total number of measured compounds is different in the 2 years (947 vs. 664 tentatively identified, respectively, Figure 4A), a large part of the ones measured in 2014 (219 of 664) was also present in 2015 (Figure 4A). Interestingly, out of the 219 common volatiles, 187 show significant differences in relative amounts in the 2 years (Wilcoxon-Mann-Withney FDR < 0.05, Supplementary Table 5, Figure 4B), and only 31 do not significantly change (Figure 4B and Supplementary Table 5). The latter ones, which can be considered “core” compound, hold the potential to be central in the definition of the Amarone organoleptic characteristics.

### Relations between mycobiota and metabolome

The identification of correlations between fungal genera abundance and quantified volatile compounds further contributes to the understanding of the changes to which Corvina grapes are subjected during the withering process. To assess if the presence of metabolites characterizing the Amarone fermentation process could be associated to a specific fungal activity, we searched for significant correlations (FDR < 0.05) among the fungal genera constantly present in the 2013 and 2014 vintages (Figure 3) and the 32 “core” Amarone compounds (Figure 4B). We carried out this analysis focusing on the “core” mycobiota and metabolome of Corvina grapes processed for Amarone production, i.e., those fungal species and compounds present in different vintages, because the identification of eventual correlations among them might have a relevant biotechnological potential. Among the fungal genera enriched in grape samples, *Phoma* showed significant positive correlations with a free compound, (3E)-3-hexenoic acid (Figure 4C). The fungal genera more abundant in musts were found to be negatively correlated with free compounds, with the only exception of *Diplodia*, which was found to be positively correlated with 1-pentanol and 2,6-dimethoxy phenol (Figure 4C). In addition, *Cytospora, Metschnikowia*, and *Candida* were found to be negatively correlated with several free compounds known to have a relevant role in wine aroma (Figure 4C and Supplementary Table 6) (Zalar et al., 2005; Gomes et al., 2013).

## Discussion

### The microbiota and metabolome of *vitis vinifera* L. cv. corvina grapes and musts are influenced by the climate and the process

The composition of the fungal microbiota present on grape skins and maintained in early phases of must fermentation has been shown to be strongly influenced by environmental factors (Bokulich et al., 2014). Nevertheless, the withering period to which Corvina grapes are subjected for Amarone production also influences the microbial populations (Salvetti et al., 2016). Here, we show that in *V. vinifera* L. cv. Corvina both the climate and the duration of withering contribute in defining the microbial populations present on grapes both at the beginning and at the end of withering period. Several parameters indicate that the fungal populations present on grapes changes only after a 7–8 weeks withering. After this period, fungal genera, despite not changing in numerosity, change in relative abundances, suggesting a selection of genera able to survive to the harsh environmental conditions imposed by the long lasting withering.

In occurrence of heavy and frequent rainfalls, wine-makers repeatedly treat vineyards with antifungal products to avoid colonization by pathogenic fungi. For this reason, the number of fungal genera present in grape and must samples could be expected to be lower in these vintages than in standard ones. Contrarily, our study revealed that samples collected during the wet vintage (2014) show a higher number of fungal genera compared to the “dry” vintage (2013), not corresponding to an increase of total amount of either bacteria or fungi. Similarly, the total amount of fungi quantified in recently harvested-grapes was higher (even if not significantly) in 2014 than in 2013. Despite the microbial quantification could be biased by the amplification of the DNA of dead cells (Nocker and Camper, 2006; Carini et al., 2016), since we observed a decrease of the total amount of fungal and bacterial DNA during the withering process we are confident that DNA does not accumulate in these samples. The fact that the total amount of fungi only slightly increased but the number of genera widely increased in wet-vintage samples compared to dry-vintage samples is ascribable to the effect of antifungal treatments in the vineyard: the killing of sensitive fungi may favor the overgrowth of otherwise not competitive fungi. The increase in number of not-sensitive fungi compensates the decrease of the sensitive ones, finally resulting in a similar total amount of fungi. The relative abundance of some fungal genera was drastically influenced by the vintage. Indeed, the relative abundance of fungal genera present prevalently either in musts (*Phomonas, Neofusicoccum, Diaporthe*, Diaporthaceae unidentified, *Davidiella, Botrytis*, and *Botrysphaeria*) or in grapes (*Nigrospora, Davidiella*, and *Wallemia*) were significantly different in the studied vintages. To note, two genera of the Botryosphaeriaceae family and known *Vitis* pathogens, *Neofusicoccum* and *Botryosphaeria*, were more abundant in 2014 than in 2013 must samples, indicating that the adverse environmental conditions could have favored their persistence on grapes even after a prolonged withering period. On the other hand, the genus *Botrytis*, found to be more abundant in 2013 than in 2014 must samples, is an example of the ability of the process to modify the environmental fungal populations. This genus is known to grow better in presence of high levels of humidity, thus potentially in the wet 2014 grapes (Paronetto and Dellaglio, 2011). Nevertheless, it has to be considered that the humidity of the warehouse used for withering was controlled and modified by an automatic system, potentially compensating for the vintage-specific environmental differences. This could explain the higher abundance of this genus in 2013 musts, composed by grapes subjected to a shorter period of withering than the 2014 grapes. Even the metabolome of Corvina is affected by the climate, since we found compounds significantly enriched in either the wet or the dry vintages. In addition, the wet-vintage samples were enriched in a higher number of compounds compared to the dry-vintage, recapitulating what observed for fungal populations, which showed a lower biodiversity in dry-vintages compared to wet-vintages. As a whole, these results indicate that either the environmental characteristics or the actions adopted to reduce the impact of environmental changes (i.e., repeated antifungal treatments and prolonged withering in presence of heavy rainfalls) can impose severe stresses resulting in a simplified microbiota adapted to this harsh environment. As a consequence of the reduced fungal biodiversity, it is likely that the ability to efficiently metabolize present compounds is reduced, finally impacting on the volatile component of the metabolome released by the microbial component (volatile compounds). As observed from the comparison of different vintages, other environmental conditions, imposed by the withering, can shape the grape mycobiota: while the initial withering does not affect the grape mycobiota, the harsh conditions imposed by a prolonged withering contribute to the shaping of the fungal populations.

### Conserved microbial and chemical characteristics of amarone fermentation

Despite the several differences observed among samples and associated to the vintage, both grapes and fresh musts of *V. vinifera* L. cv. Corvina showed fungal genera which were constantly present in different years. As expected, the genera characterizing the fungal mycobiota of Corvina grapes are those usually found in the environment, confirming the environmental origin of the microbial populations inhabiting the grape skin (Barata et al., 2011). Among the fungal genera whose relative abundances remain unchanged during different vintages, Leptosphaerulina, *Pithomyces*, and *Phoma* are commonly associated to plants and soil, while *Alternaria* and *Cryptococcus* are commonly found associated to fruits and vegetables. Even more important, several fungal genera among those constantly present in must samples of different vintages, like *Zygosaccharomyces, Metschnikowia*, and *Candida*, are known to hold relevant roles (either useful or detrimental) in the fermentation for the production of volatile aroma compounds (Loureiro and Malfeito-Ferreira, 2003; Sipiczki, 2006; Oro et al., 2014; Whitener et al., 2015, 2016; Ciani et al., 2016). Interestingly, several negative correlations were found among fungal genera more abundant in grapes and genera more abundant in musts. Such negative correlations could indicate either the presence of competition among genera or the inability of the two genera to exploit the same environment. To note, in a recent study Salvetti et al. found some fungal genera at the end of the *V. vinifera* L. cv. Corvina grapes withering (i.e., *Aspergillus* and *Penicillium*) which we did not find in either grapes or fresh musts in the studied vintages (Salvetti et al., 2016). These known airborne molds mainly grow in humid and warm environments. The use of temperature and humidity controller systems in the warehouse used for withering the grapes could have prevented the growth of these molds.

Interestingly, we found that *Diplodia* (found exclusively in must samples) was positively correlated with compounds usually detected in traces in grapes and fresh fruits, 1- pentanol and 2,6-dimethoxyphenol (the latter also called syringol) (Escudero et al., 2007). These compounds increase during grapes withering, confirming previous observations for compounds of the same class (Bellincontro et al., 2016). We also found that octanoic acid (caprylic acid) had a negative correlation with fungal genera increasing in relative abundance during the withering process, indicating a decrease of the compound during the withering. Considering that this compound is known to inhibit *Saccharomyces cerevisiae* fermentation (Stevens and Hofmeyr, 1993), by subjecting the grapes to a prolonged withering process the protocol for Amarone production could favor the growth of this yeast in the following fermentation.

## Concluding remarks

As all the other fermentative processes, also Amarone production is strongly affected by the environmental conditions during grapes maturation, thus by the climate. Our findings highlight that, despite some changes that could be ascribed to the vintage, both microbial populations and chemical profile of *V. vinifera* L. cv. Corvina grapes and musts show characteristics that are maintained over different vintages. These characteristics, either defined by the process or by chemico-physical parameters (i.e., of the Corvina cultivar, of the soil), contributes to the typicity of Amarone. The disclosure of these persistent microbial and chemical features helps in understanding the details of this production technology and will thus give pivotal insights to further optimize the process.

## Author contributions

DC, IS, and FM conceived and designed the study. IS, MP, and NT handled the samples and carried out DNA extractions, qRT-PCR, and PCR amplification before library preparation. AZ, ST, and AB were responsible for the processes conducted in the winery. SC, UV, NT, and FM extracted and analyzed the metabolites. PF supervised and carried out statistical analyses. CD and DA supervised and carried out metataxonomics analyses. All the authors discussed the results and contributed at writing the manuscript.

## Funding

This study was supported by Cantina Zenato.

### Conflict of interest statement

The authors declare that the research was conducted in the absence of any commercial or financial relationships that could be construed as a potential conflict of interest.
