# Supplementary material for: Core Microbiota and Metabolome of Vitis vinifera L. cv. Corvina Grapes and Musts
Source: Front Microbiol. 2017 Mar 21;8:457. doi: 10.3389/fmicb.2017.00457 (PMC5359246; doi:10.3389/fmicb.2017.00457)
Supplement: Supplementary file 7 [file SupplementaryImages.PDF]

Supplementary Material

Core microbiota and metabolome of *Vitis vinifera* L. cv. Corvina Grapes and Musts

Irene Stefanini, Silvia Carlin, Noemi Tocci, Davide Albanese, Claudio Donati,Pietro Franceschi, Michele Paris, Alberto Zenato, Silvano Tempesta, Alberto Bronzato, Urska Vrhovsek, Fulvio Mattivi*, Duccio Cavalieri

*** Correspondence:** Corresponding Author: ulvio.mattivi@unitn.it

Supplementary Figures:

Supplementary Figure 1: Alpha diversities of samples.

Supplementary Figure 2: Beta diversities of samples.

Supplementary Figure 3: Welch statistics comparing the relative abundances of fungal genera present in grapes sampled at T0 and T1 in the two vintages.

Supplementary Figure 4: Welch statistics comparing the relative abundances of fungal genera present in the two vintages.

Supplementary Figure 5: Relative abundances of fungal genera.

Supplementary Figure 6: Phylogenetic tree of Candida and Metschnikowia spp.

Supplementary Figure 7: Pearson correlations among fungal genera significantly more abundant in either must or grape samples.

Other Supplementary materials (available as separate .xlsx files):

Supplementary Table 1: Summary of the results of BLAST analysis carried out on the representative sequences through the National Center for Biotechnology Information nucleotide collection database (http://blast.ncbi.nlm.nih.gov/Blast.cgi?PROGRAM=blastn&PAGE_TYPE=Blast Search&LINK_LOC = blasthome)

Supplementary Table 2: Wilcoxon-Mann-Whitney test on microbial alpha and beta diversities.

Supplementary Table 3: Results of Wilcoxon-Mann-Whitney tests comparing the relative abundances of volatile compounds in grape (T1) and must samples.

Supplementary Table 4: Results of Wilcoxon-Mann-Whitney tests comparing the relative abundances of glycosylated precursor compounds in grape (T1) and must samples.

Supplementary Table 5: Results of Wilcoxon-Mann-Whitney tests comparing the relative abundances of volatile compounds in samples of the two studied vintages.

Supplementary Table 6: Spearman correlations between "core" fungal genera and volatile compounds (not changing in relative abundance among vintages, as shown by Wilcoxon-Mann-Whitney results).Supplementary Figures

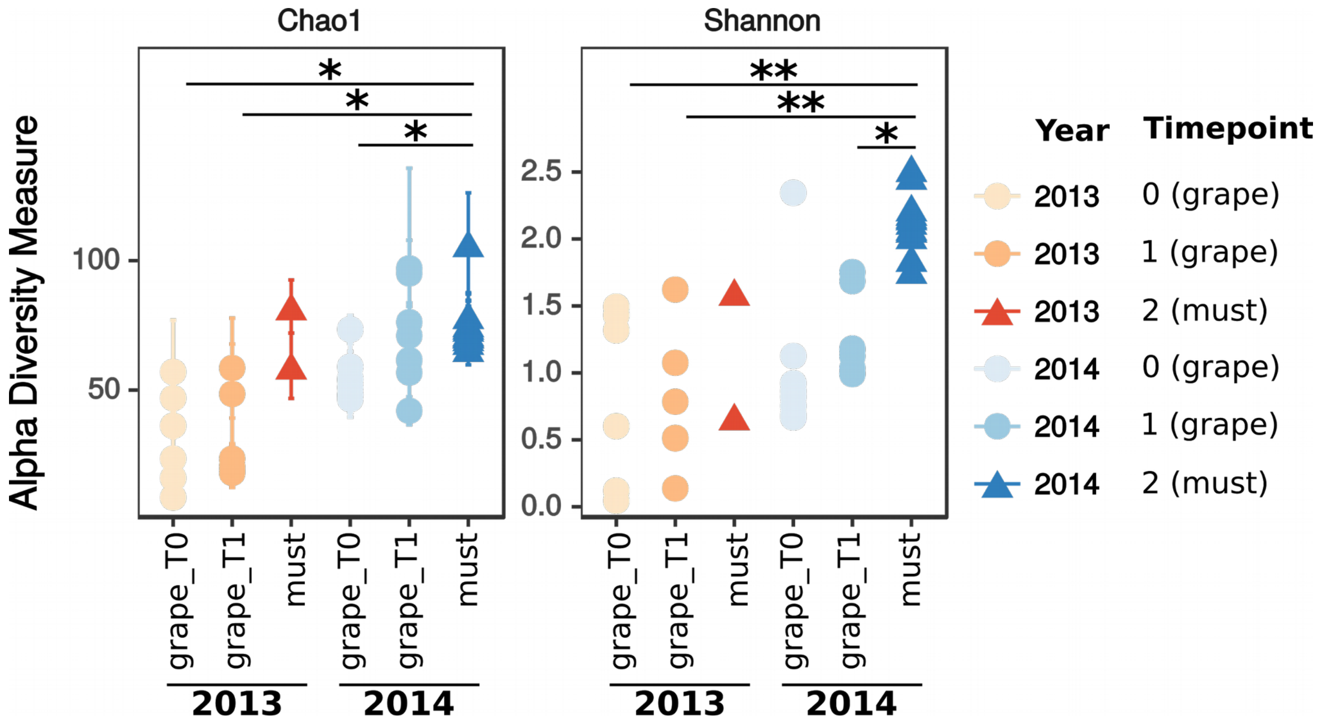

Supplementary Figure 1: Alpha diversities of samples. Alpha diversity of samples calculated with the Chao method and the Shannon index.

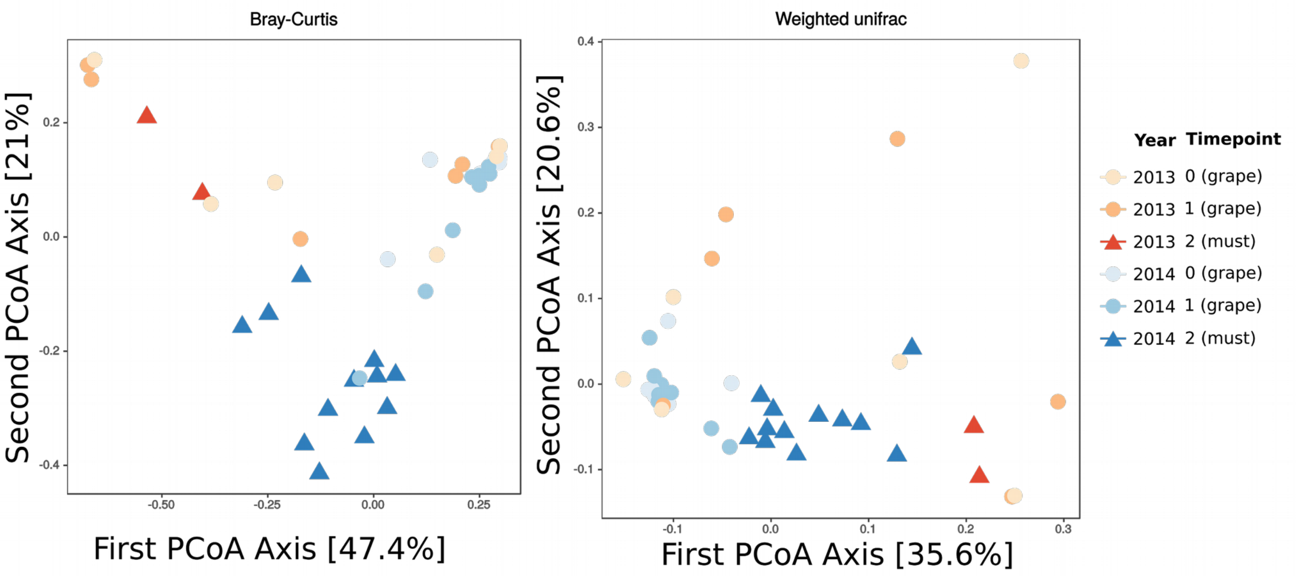

Supplementary Figure 2: **Beta diversities of samples. a-** Principal Coordinate Analysis on Bray-Curtis beta diversities among samples. **b-** Principal Coordinate Analysis on unweighted unifrac beta diversities among samples.

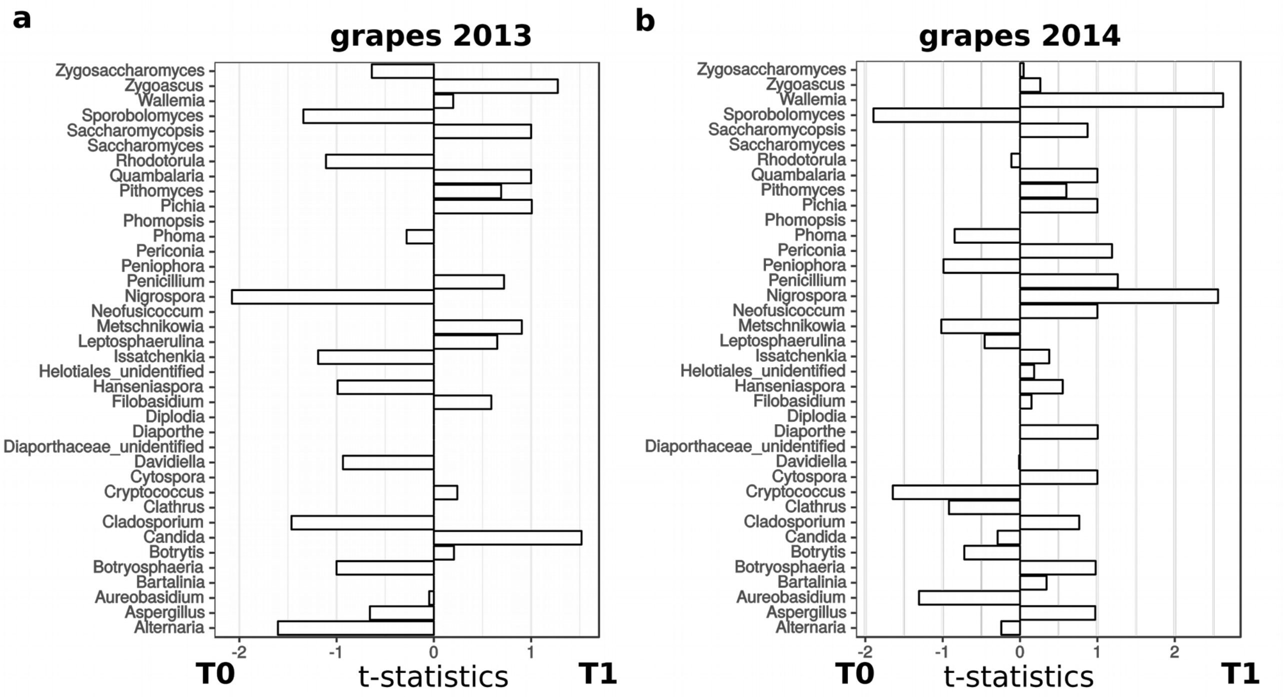

<u>Supplementary Figure 3</u>: Welch statistics comparing the relative abundances of fungal genera present in grapes sampled at T0 and T1 in the two vintages. a- comparison of genera relative abundances in 2013 grape samples. **b-** comparison of genera relative abundances in 2014 grape samples. Welch test was carried out to compare the relative abundances of fungal genera in grape and must samples. White= Welch t-test FDR>0.05.

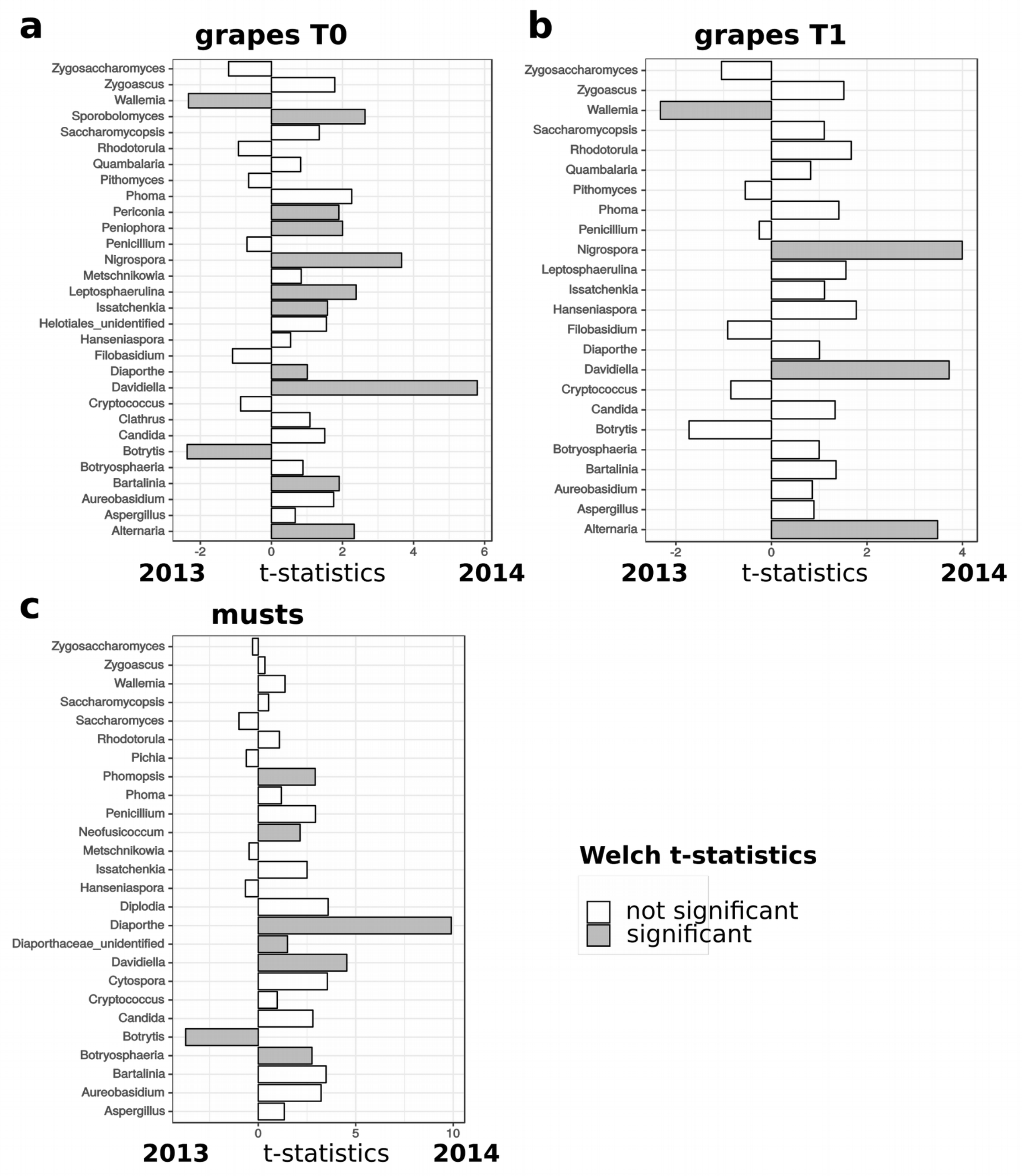

Supplementary Figure 4: Welch statistics comparing the relative abundances of fungal genera present in the two vintages. a- genera relative abundances in grape sampled at T0. **b-** genera relative abundances in grape sampled at T1. **c-** genera relative abundances in must samples. Welch test was carried out to compare the relative abundances of fungal genera in the two vintages. Grey=Welch t-test among vintages p<0.05, white= Welch t-test p>0.05. Only genera with total relative abundance >1% are shown.

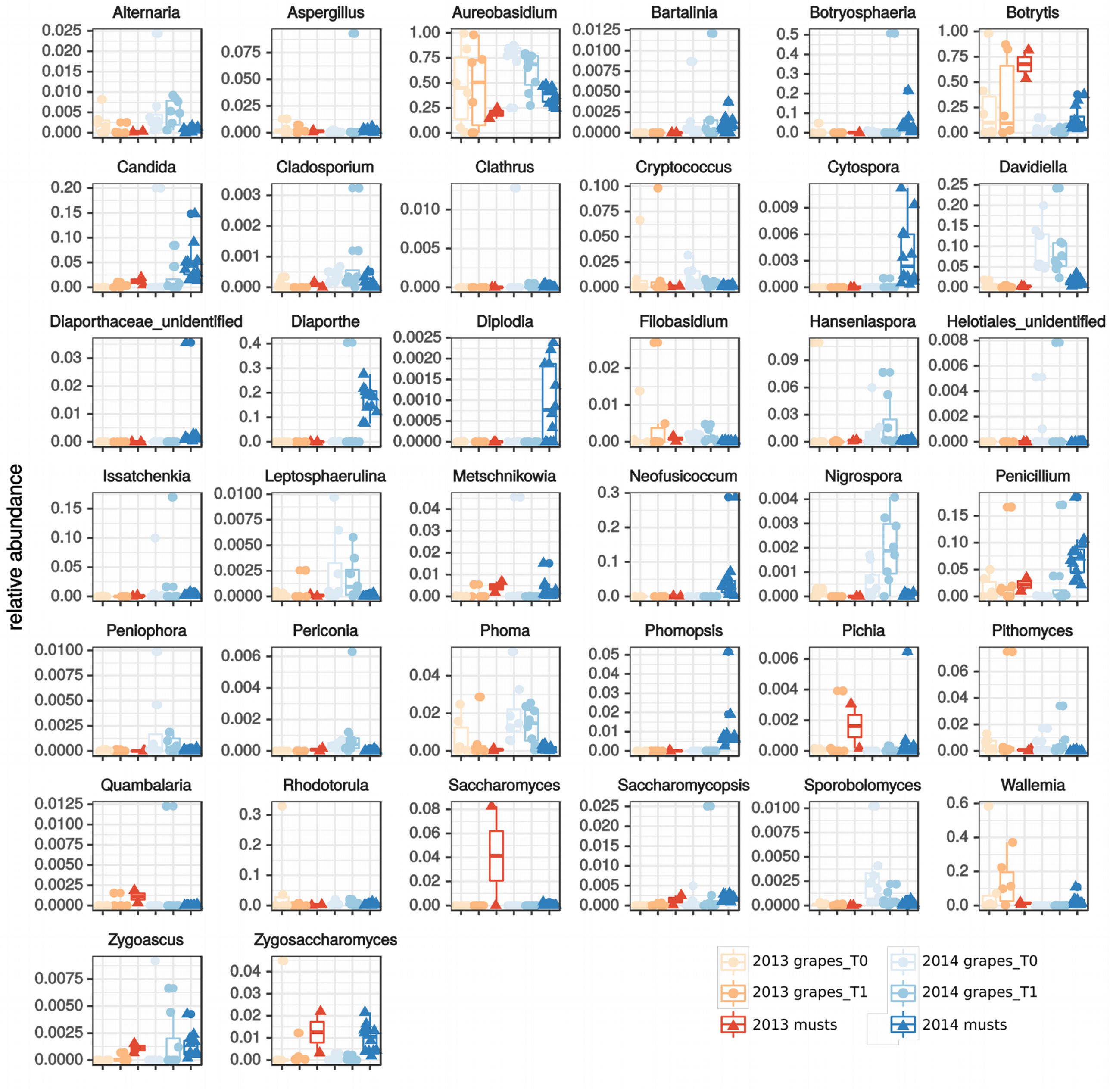

Supplementary Figure 5: Relative abundances of fungal genera.

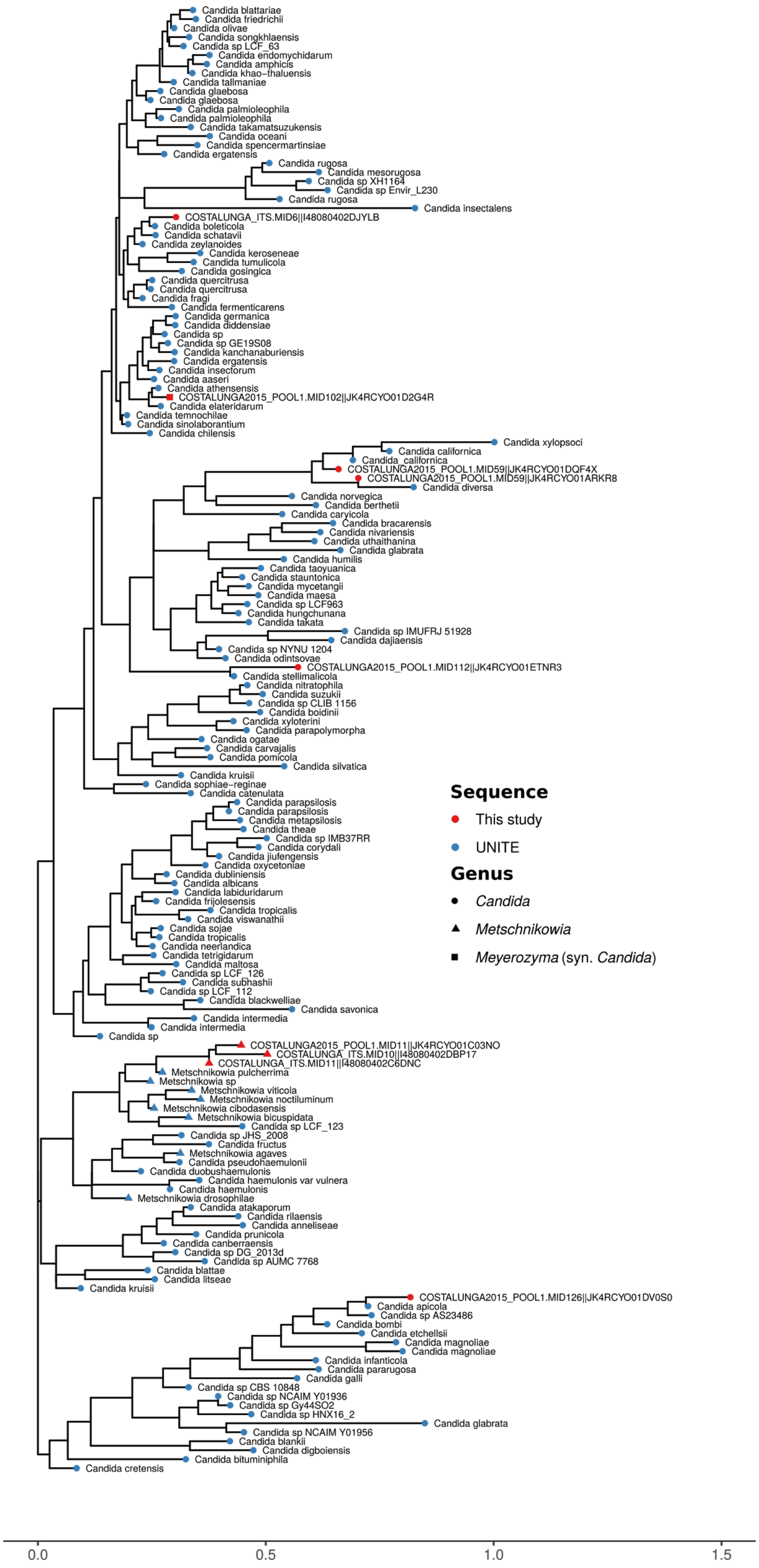

Supplementary Figure 6: Phylogenetic tree of *Candida* and *Metschnikowia* spp. Maximum-likelihood build on the T-Coffee alignment of the OTUs identified as belonging to the *Candida* and *Metschnikowia* genera and the reference Unite database corresponding to all the species of these genera.

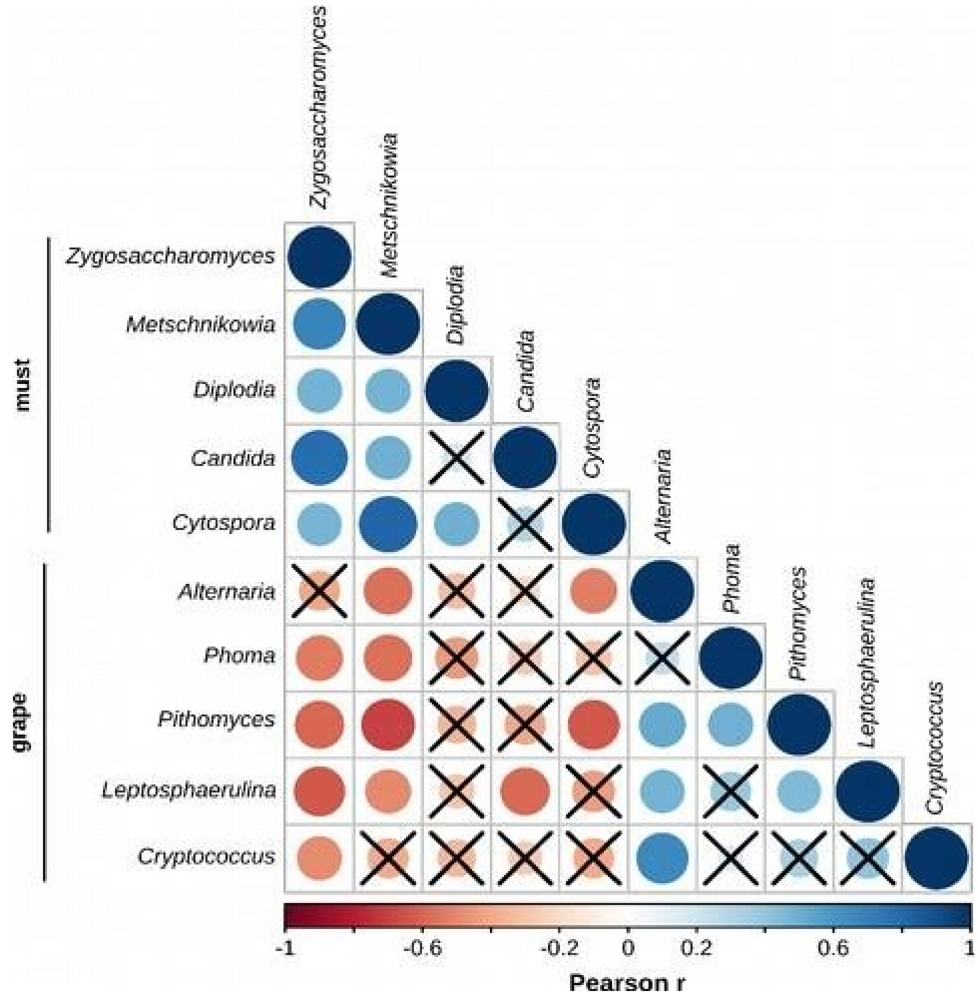

[Supplementary Figure 7:](#) **Pearson correlations among fungal genera significantly more abundant in either must or grape samples.** Dots radius is proportional to the Pearson correlation r. The dots color and size indicate the Pearson r, as shown in the legend below the matrix. Crossed dots have p>0.05. Lateral “must” and “grape” labels are only indicative, to show the genera which were found to be more represented in one of the two specimen.
